# Beta-Klotho Protein Expression in Healthy Human Tissues and Liver Biopsies From Patients With MASLD or MASH

**DOI:** 10.1016/j.gastha.2025.100745

**Published:** 2025-07-11

**Authors:** Alexandra S. Aaldijk, Dicky Struik, Stan Driessen, Cristy R.C. Verzijl, Joanne Verheij, Max Nieuwdorp, Roos Eilers, Justina C. Wolters, Adriaan G. Holleboom, Johan W. Jonker

**Affiliations:** 1 Department of Pediatrics, University of Groningen, University Medical Center Groningen, Groningen, The Netherlands; 2 Department of Vascular and Internal Medicine, Amsterdam University Medical Center, Amsterdam, The Netherlands; 3 Department of Pathology, Amsterdam University Medical Center, Amsterdam, The Netherlands; 4 Interfaculty Mass Spectrometry Center, University of Groningen, University Medical Center Groningen, Groningen, The Netherlands

**Keywords:** FGF19, FGF21, MASLD, Hepatic Steatosis, Inflammation

## Abstract

**Background and Aims:**

Biologics based on the structure of fibroblast growth factor (FGF) 19 and 21 show strong beneficial effects in the treatment of metabolic dysfunction–associated steatotic liver disease (MASLD), including compensated cirrhosis. Studies in rodents indicated that the effectiveness of these drugs relies on the presence of transmembrane protein beta-klotho (KLB). However, the tissue expression profile of KLB and its regulation in liver disease remain poorly characterized. Here, we aim to investigate KLB protein expression in healthy human tissues and liver biopsies from patients with MASLD.

**Methods:**

Following extensive antibody validation, immunohistochemical analyses were conducted on paraffin-embedded human tissue samples to determine KLB protein tissue distribution. Subcellular localization of KLB was examined in cell lines expressing KLB either ectopically or endogenously. Additionally, KLB protein levels were quantified in 28 liver biopsies from patients with MASLD within the ANCHOR cohort study and correlated with histological MASLD features and clinical patient characteristics.

**Results:**

KLB protein expression was observed in the liver, bile ducts, gallbladder, stomach, colon, adipose tissue, and pancreas. Localization studies revealed that KLB was predominantly localized to the plasma membrane in both ectopic and endogenous contexts. KLB was also detected in liver biopsies from patients with MASLD/metabolic dysfunction–associated steatohepatitis and remained expressed at advanced stages of MASLD. Lower levels of hepatic KLB protein were significantly associated with higher levels of lobular inflammation (*P* = .0168) but not with histology- or magnetic resonance imaging–derived scores of steatosis or fibrosis.

**Conclusion:**

This study provides insight into target organs for FGF-based drugs and demonstrates that hepatic KLB remains expressed throughout MASLD stages, supporting the use of FGF-based drugs in early and advanced stages of MASLD.

## Graphical abstract

**Figure:**
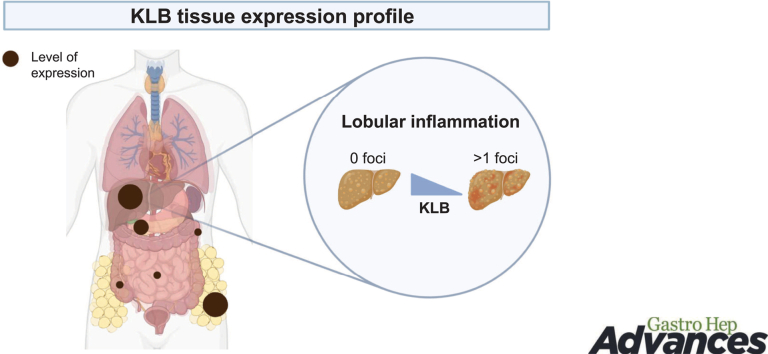

## Introduction

Metabolic dysfunction–associated steatotic liver disease (MASLD) and its advanced subtype, metabolic dysfunction–associated steatohepatitis (MASH), are rapidly rising global health concerns. The prevalence of MASLD is steadily increasing and MASH is a leading cause of liver-related morbidity and mortality.^1^ The spectrum of MASLD ranges from isolated steatosis, characterized by greater than 5% hepatic lipid accumulation, which can advance to MASH, which also encompasses hepatocellular damage and inflammation, and ultimately to liver fibrosis, cirrhosis, and hepatocellular carcinoma.^2^ Both hepatic inflammation and fibrosis have been described as key drivers influencing mid-term and long-term mortality in patients with MASLD.^3^ Recently, resmetirom, a selective thyroid hormone receptor-beta agonist, was the first drug in the USA to obtain Food and Drug Administration approval for the treatment of MASH with moderate to advanced fibrosis.^4^ While this is a significant breakthrough, resmetirom does not improve fibrosis in all patients, highlighting the need to develop additional therapeutic strategies.

Fibroblast growth factor (FGF) 19 and 21–based biologics are also highlighted as potential treatments for MASLD due to their strong beneficial effects on metabolism.^5^^,^^6^ FGF19 and FGF21 are endocrine members of the FGF family and crucial in regulating bile acid, glucose, and lipid metabolism.^7^ FGF19 is synthesized in the ileum in response to bile acid absorption and acts by inhibiting bile acid synthesis in the liver.^8^ FGF21 is primarily synthesized in the liver in response to metabolic stress and broadly affects glucose and lipid metabolism, primarily via its actions on adipose tissue.^9^ Due to these strong metabolic effects, FGF19- and FGF21-mimetics were developed and tested for their effectiveness in treating metabolic diseases. Notably, the most pronounced clinical improvements have been observed in the treatment of MASLD.^10^ Treatment with FGF19 analog NGM282 led to metabolic improvement of MASH in animal models, showing ameliorated bile acid toxicity, decreased hepatic inflammation, fibrosis, and lipid content.^11^^,^^12^ In phase 2 human clinical trials, 2 weeks of NGM282 treatment diminished liver fat content and improved liver damage markers and histological features of MASH.^13^^,^^14^ Prolonged treatment also decreased plasma C4 levels and liver fibrosis biomarker pro-C3.^15^ Treatment with the polyethylene glycol–conjugated FGF21 analog Pegbelfermin has also shown promising results in patients with MASH, namely a decrease in hepatic fat fraction and improvements in liver stiffness, biomarkers of liver damage, and fibrosis.^16^ Another promising FGF21-based drug, Etruxifermin, showed a reduction in hepatic fat and NASH resolution without worsening fibrosis as well as a significant reduction in enhanced liver fibrosis scores.^17^^,^^18^ A recent meta-analysis also shows that the resolution of MASH and fibrosis by FGF21 analogs is superior to resmetirom and glucagon-like peptide 1 analogs.^19^

Rodent studies have demonstrated that the metabolic improvements after FGF19 or FGF21 administration depend on the FGF coreceptor beta-klotho (KLB).^20^^,^^21^ The KLB gene was initially discovered based on its similarity to the Klotho gene, which is implicated in phosphate metabolism and aging processes.^22^^,^^23^ However, studies on KLB have highlighted different metabolic effects compared to the Klotho gene. *Klb*-deficient mice exhibit increased bile acid synthesis and excretion due to increased expression of cholesterol 7α-hydroxylase, the rate-limiting enzyme in bile acid synthesis.^24^^,^^25^ Similarities between *Klb*-deficient and *Fgf15*-and *Fgfr4*-deficient mice,^26^^,^^27^ along with subsequent molecular studies,^28^^,^^29^ have established KLB as an obligatory coreceptor for murine FGF15, its human ortholog FGF19, and FGF21 in activating FGF receptors.

Additionally, genetic studies have linked the KLB variant rs17618244 to increased lobular inflammation, ballooning, and fibrosis, implicating a role of KLB in MASLD development.^30^^,^^31^ Mechanistic studies in rodents indicate that KLB-expressing tissues, such as the liver, adipose tissue, and certain brain areas, play a crucial role in driving the metabolic effects of FGFs.^20^^,^^21^ Despite the pivotal role of KLB in the action of FGF-based therapies, its expression and localization in human tissues and cells remain relatively unexplored. Additionally, the impact of MASLD on hepatic KLB expression is unclear, which is vital for understanding the therapeutic potential and limitations of targeting KLB.

To address these knowledge gaps, we systematically mapped KLB protein expression across healthy human tissues and investigated its subcellular localization. Additionally, we determined whether MASLD progression affects KLB expression levels, potentially influencing the effectiveness of FGF-based treatments. Our findings provide crucial insights into the potential target organs for FGF-based drugs and the viability of KLB-targeting therapies at different stages of MASLD.

## Methods

### Tissues and Reagents

Paraffin-embedded human and monkey tissue sections, and human tissue protein lysates were obtained from the Biochain Institute (Table A1-3). Primary and secondary antibodies AB1 (106794, Abcam), AB2 (SAB2108630, Sigma Aldrich), AB3 (MAB5889, R&D systems), and AB4 (AF5889, R&D systems) for KLB were used for antibody validation. Expression of KLB was established using a plasmid containing human KLB (OHu24417, GenScript). Recombinant human KLB (5889-KB), recombinant cynomolgus monkey KLB (10428-KB), recombinant mouse KLB (2619-KB), recombinant human klotho (5334-KL), recombinant human cytosolic beta-glucosidase/GBA3 (5969-GH), and recombinant human FGF21 (2539-FG) were obtained from R&D Systems.

### Human MASLD Liver Biopsies

KLB protein levels in liver tissue were quantified in 28 histological specimens of the ANCHOR cohort. The Amsterdam MASLD/MASH cohort (ANCHOR) study is an ongoing observational prospective double-biopsy study aimed at determining the natural progression of MASLD and identifying and validating both imaging and molecular markers for the assessment of the MASLD spectrum.^32^ Further details of the ANCHOR study are reported in Troelstra et al. (2021). Biopsies of male and female patients were included in this study to ensure an adequate representation of MASLD patients.

Percutaneous ultrasound-guided liver biopsies were performed by an interventional radiologist or a hepatologist according to local standard procedure. Histological scoring was done by 2 expert liver pathologists who were blinded to all other data. Biopsy samples used for histological scoring were stained with hematoxylin and eosin, Sirius Red, and others. Histological parameters were defined using the steatosis, activity, and fibrosis score as previously reported.^33^ MASLD was defined by the presence of steatosis in at least 5% of hepatocytes. MASH was diagnosed in cases with each of the following: steatosis, hepatocellular ballooning grade of at least 1, and lobular inflammation grade of at least 1. Histological characteristics were scored according to the NASH Clinical Research Network as follows: F0: no fibrosis, F1: perisinusoidal or periportal fibrosis, F2: perisinusoidal and periportal fibrosis without bridging, F3: bridging fibrosis, and F4: cirrhosis.^34^ Steatosis was assessed using a scale from 0 to 3: 0 (<5%), 1 (5%–33%), 2 (34%–66%), and 3 (>67%). Hepatocellular ballooning was graded on a scale of 0–2: 0 (normal hepatocytes), 1 (clusters of hepatocytes with a rounded shape and pale cytoplasm, but of normal size), and 2 (as in grade 1, but with at least one enlarged or ballooned hepatocyte). Lobular inflammation was defined as a focus of 2 or more inflammatory cells within the lobule and was graded as follows: 0 (none), 1 (≤2 foci per lobule), or 2 (>2 foci per lobule).

For magnetic resonance imaging (MRI) analysis of patients with MASLD, see Supplementary Methods.

### Immunohistochemistry

Tissue sections were deparaffinized in xylene and rehydrated through a series of ethanol washes (100%–50%). Antigen retrieval was performed by boiling sections for 20 minutes in 10 mM citrate buffer (pH 6.0) for KLB and insulin staining or 10 mM Tris-ethylenediaminetetraacetic acid buffer (pH 9.0) for cytokeratin 19 (CK19) staining. After washing, sections were blocked for 30 minutes with 3% hydrogen peroxide for KLB and CK19 staining or 0.3% hydrogen peroxide for insulin staining. Sections were incubated with 5% bovine serum albumin (BSA) for KLB, 1% BSA for insulin, or 5% goat serum for CK19 in a humidified chamber for 30 minutes. Next, sections were incubated overnight at 4 °C with 1:100 primary KLB (AF5889, R&D systems), 1:1000 CK19 (ab52625, Abcam), or 1:500 insulin antibody (A0564, Dako). The following day, sections were washed and incubated at room temperature (RT) with 1:100 horseradish peroxidase (HRP)-conjugated rabbit antigoat immunoglobulin G (IgG) antibody (P0449, Dako), 1:100 HRP-conjugated goat antirabbit IgG (P0448, Dako), or 1:200 HRP-conjugated rabbit anti-guinea pig IgG (A5545, Sigma). Insulin staining required an additional 60-minute incubation at RT with a 1:200 dilution of tertiary goat anti-rabbit antibody (P0448, Dako) in 1% BSA. All sections were then incubated with 3, 3-diaminobenzidine (SK-4100, Vector Laboratories) for 10 minutes at RT and counterstained with hematoxylin for 1 minute. Sections were washed with tap water and rehydrated through a graded ethanol series. Finally, sections were sealed with Eukitt Quick-hardening mounting medium (03989, Sigma Aldrich), and quantified using the Aperio ImageScope software.

For experiments on cell lines (lentiviral shRNA knockdown, siRNA-based transfection, subcellular fractionation, fluorescence-activated cell sorting (FACS), immunofluorescence (IF), quantitative polymerase chain reaction, immunoblotting, targeted proteomics), see Supplementary Methods.

### Statistical Analysis

Statistical analyses were performed using GraphPad Prism 10.2.3. Software. Nonparametric Kruskal–Wallis testing was performed to determine statistical differences in hepatic KLB levels between different histological groups. Spearman’s rank test was performed to identify correlations between hepatic KLB levels and clinical patient characteristics, followed by Benjamini–Hochberg correction for multiple testing. Data are presented as median ± interquartile range in individual value plots. Significance was indicated as ∗*P* < .05, ∗∗*P* < .01, or ∗∗∗*P* < .001.

## Results

### KLB Antibody Validation

To identify antibodies that are specific and selective for KLB, we evaluated the ability of four commercially available antibodies (AB1-4) to detect recombinant or endogenous KLB using previously established guidelines for antibody validation.^35^^,^^36^ Using immunoblotting, all 4 antibodies detected recombinant KLB, reflected by a single band between 100 and 150 kDa (Figure A1) in a dose-dependent manner (Figure A2), indicating correct epitope recognition. Next, we assessed the cross-reactivity of AB1-4 against orthologous and paralogous proteins. AB1–AB4 showed cross-reactivity with cynomolgus monkey KLB (97% amino acid similarity) and mouse KLB (79% amino acid similarity) but not with human klotho (46% amino acid similarity) or human glucosylceramidase beta 3 (38% amino acid similarity) (Figure A3).

Next, we assessed antibody selectivity by spiking recombinant human KLB into a lysate of HEK293 cells lacking endogenous KLB expression (normalized transcripts per million (nTPM) of 0.1).^37^ In these spiked lysates, AB1, AB3, and AB4 detected a strong band between 100 and 150 kDa, whereas AB2 also detected a strong band between 25 and 37 kDa (Figure A4). We also evaluated antibody selectivity in cell lines that express KLB endogenously, including HepG2 and Hep3B, and compared this to KLB-deficient HEK293 cells. Endogenous KLB expression in these cell lines was confirmed using quantitative PCR and targeted proteomics (Figure A5A–C). While AB1 did detect a band of the anticipated molecular weight (100–150 kDa) in cell lines expressing KLB endogenously, it also detected a band of the same size in KLB-deficient HEK293 cells (Figure. A5C). In contrast, AB2 and AB3 did not detect a band at the anticipated molecular weight (100–150 kDa) in any cell line (Figure A5C). Only AB4 detected a band at the anticipated molecular weight in all cell lines expressing KLB endogenously, while this band was absent in KLB-deficient HEK293 cells (Figure A5C). Based on these findings, we continued our validation process using AB4.

Although AB4 detected a band of the expected molecular weight in KLB-expressing cell lines, cross-reactivity with proteins of similar weight is still possible. Upon siRNA-mediated knockdown of KLB in Hep3B cells, detection of the 100–150 kDa band was largely lost, showing that AB4 recognizes endogenous KLB and does not cross-react with proteins of a similar molecular weight (Figure A6A). Conversely, after overexpression of human KLB in KLB-deficient HEK293 cells, AB4 detected a 100–150 kDa band with increased intensity after prolonged overexpression (Figure A6B).

**Figure 1:**
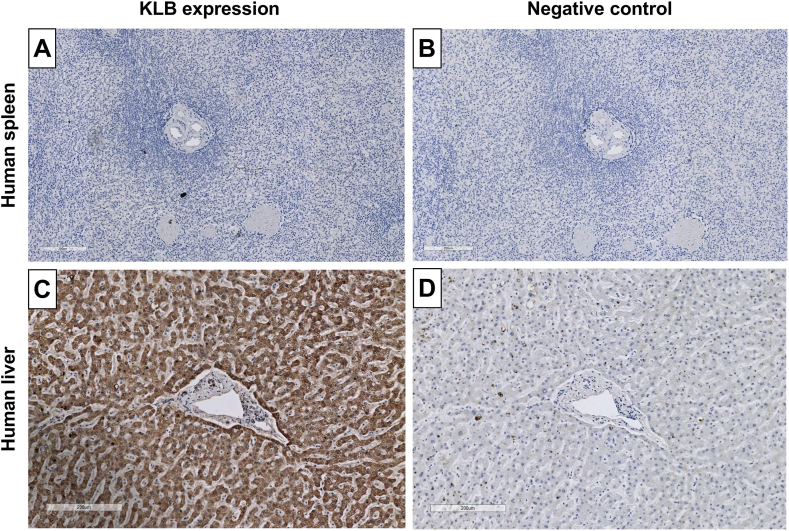
Validation of AB4 for IHC using human spleen (KLB-negative) and liver tissue (KLB-positive). KLB protein expression in (A) human spleen tissue with (B) negative control and (C) human liver tissue with (D) negative control. Scale bars represent 200 μm.

To further validate the antibody for immunohistochemistry, we leveraged the fact that KLB expression is restricted to a limited number of tissues. According to publicly available mRNA expression datasets (https://proteinatlas.org), KLB mRNA is highly expressed in the liver (nTPM 12.2–17.2), specifically in hepatocytes (nTPM 43.0), while there is no evidence for KLB expression in the spleen (nTPM 0.0)^37^, ^[dataset]^.^38^ Using this orthogonal antibody validation strategy, we observed that AB4 specifically stained hepatocytes in the human liver (Figure 1C), while no staining was observed in the spleen (Figure 1A) or tissues stained with only the secondary antibody (Figure 1B and D). Similarly, AB4 detected a strong 100–150 kDa band in a protein lysate of a healthy human liver, while this band was not detected in the human spleen (Figure A7). Collectively, our validation efforts identify AB4 as the most suitable antibody to detect KLB in cells or tissues using immunoblotting or IHC.

### KLB Protein Levels in Human Tissues

**Figure 2:**
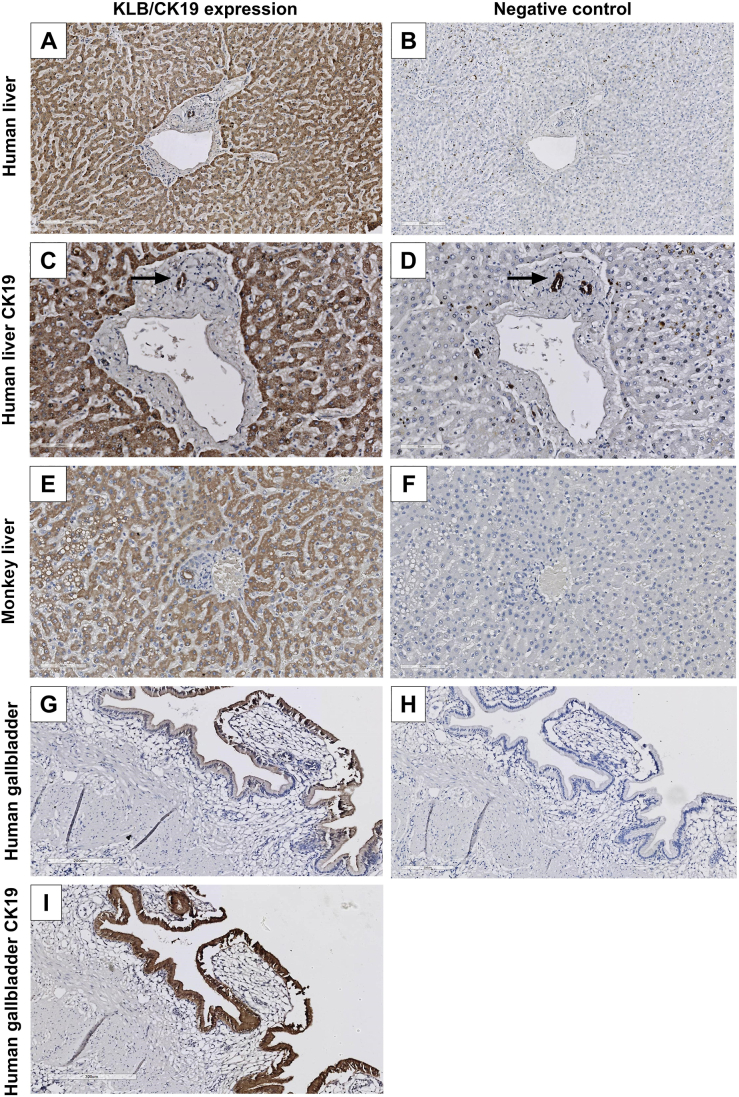
KLB protein expression levels using AB4 and CK19 in human and monkey tissues. KLB protein expression levels in (A) human liver (200 μm) with (B) negative control (200 μm), (C) human bile ducts in liver (80 μm) with (D) CK19 staining as positive control (80 μm), (E) monkey liver (90 μm) with (F) negative control (90 μm), (G) human gallbladder (200 μm) with (H) negative control (200 μm) and (I) CK19 staining as positive control (300 μm).

To systematically investigate KLB expression in healthy human tissues, we selected tissues showing evidence of KLB mRNA expression (nTPM>1.0) based on publicly available RNA expression datasets,^37^ ^[dataset]^.^38^ Tissues with KLB mRNA expression included liver, adipose tissue, pancreas, mammary gland, testis, lung, stomach, and colon. Immunohistochemical analysis of these tissues revealed strong KLB protein expression in hepatocytes and cholangiocytes in the liver (Figure 1C, Figure 2A and 2C). Specific expression of KLB in bile ducts was confirmed by additional staining with the cholangiocyte marker CK19 (Figure 2D). No KLB staining was seen in sinusoidal endothelial cells or other cell types in the portal triad. A similar expression pattern of KLB in hepatocytes and bile ducts was observed using IF (Figure A8A–B). However, we could not distinguish between cytoplasmic and membrane localization. Given that AB4 demonstrated strong cross-reactivity with cynomolgus monkey KLB (Figure A3), we sought to replicate these findings in liver tissue of cynomolgus monkey, a model organism frequently used to study FGF-based drug activity.^39^^,^^40^ Consistent with the human liver, KLB expression was observed specifically in the hepatocytes and cholangiocytes of monkey liver tissue (Figure 2E–F). As cholangiocytes also line the gallbladder, we performed IHC on human gallbladder mucosa sections, showing specific KLB expression in cholangiocytes (Figure 2G–H). A similar expression pattern was seen after staining for CK19 (Figure 2I). While KLB expression was detected in both the cytoplasm and membrane, the staining intensity was more pronounced in the membrane region. More pronounced KLB membrane expression was also observed after immunofluorescent staining of gallbladder sections (Figure 2G, Figure A8C–D).

**Figure 3:**
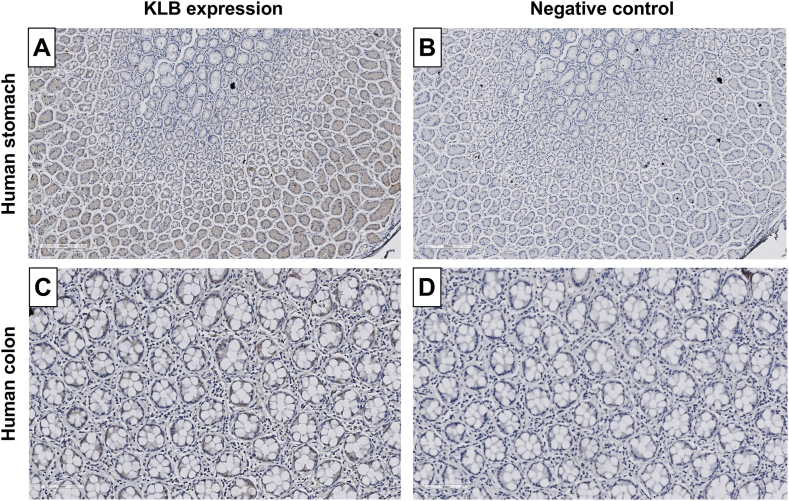
KLB protein expression levels using AB4 along the human gastrointestinal tract. KLB protein expression levels in (A) human stomach (200 μm) with (B) negative control (200 μm), and (C) human colon tissue (90 μm) with (D) negative control (90 μm).

Next, we examined KLB expression along the gastrointestinal tract. Although bulk tissue mRNA expression data only showed evidence of KLB expression in the stomach, single-cell expression data also suggested specific enrichment of KLB in intestinal goblet cells.^37^ Hence, KLB expression in the small intestine and colon was also examined. In the stomach, KLB expression was specifically detected in the gastric glands (Figure 3A–B). In the colon, weak expression of KLB was observed in the intestinal glands, while no KLB expression could be detected in the small intestine (Figure 3C–D, Figure A9A–D).

**Figure 4:**
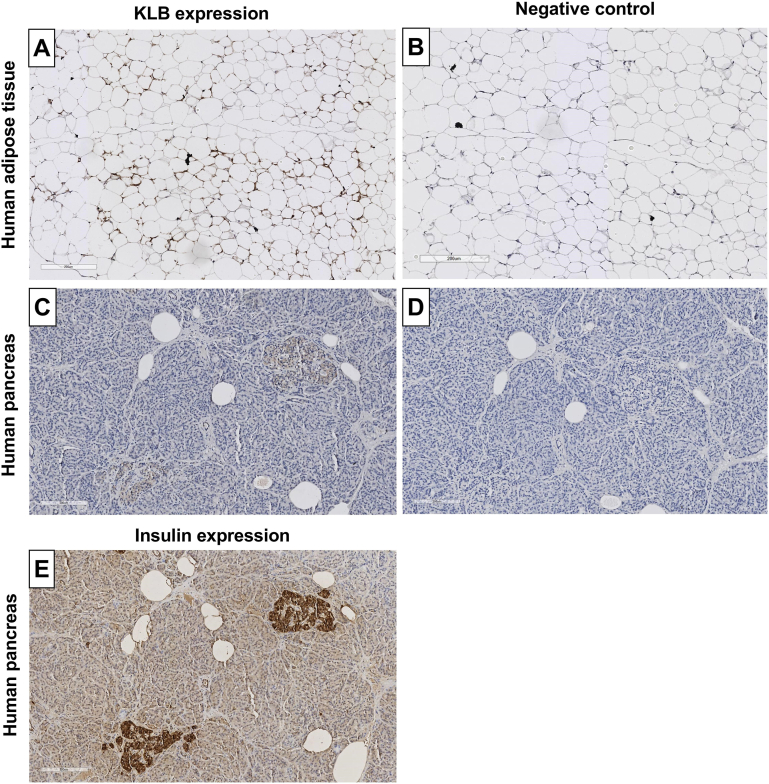
KLB protein expression levels using AB4 in human adipose tissue and pancreas. KLB protein expression levels in (A) human adipose tissue with (B) negative control, and (C) human pancreas with (D) negative control and (E) insulin staining as positive control to visualize the islets of Langerhans. Scale bars represent 200 μm.

Evident KLB expression was also observed in the adipose tissue and pancreas (Figure 4A–D). In the pancreas, KLB appeared to be expressed explicitly within the pancreatic islets of Langerhans, which was confirmed by costaining with insulin (Figure 4E). IF staining on adipose tissue sections suggested both cytoplasmic and membrane localization (Figure A8E–F). No clear KLB expression was observed in the mammary gland, lung, and testis (Figure A9E–J).

### Subcellular Localization of Ectopically and Endogenously Expressed KLB

**Figure 5:**
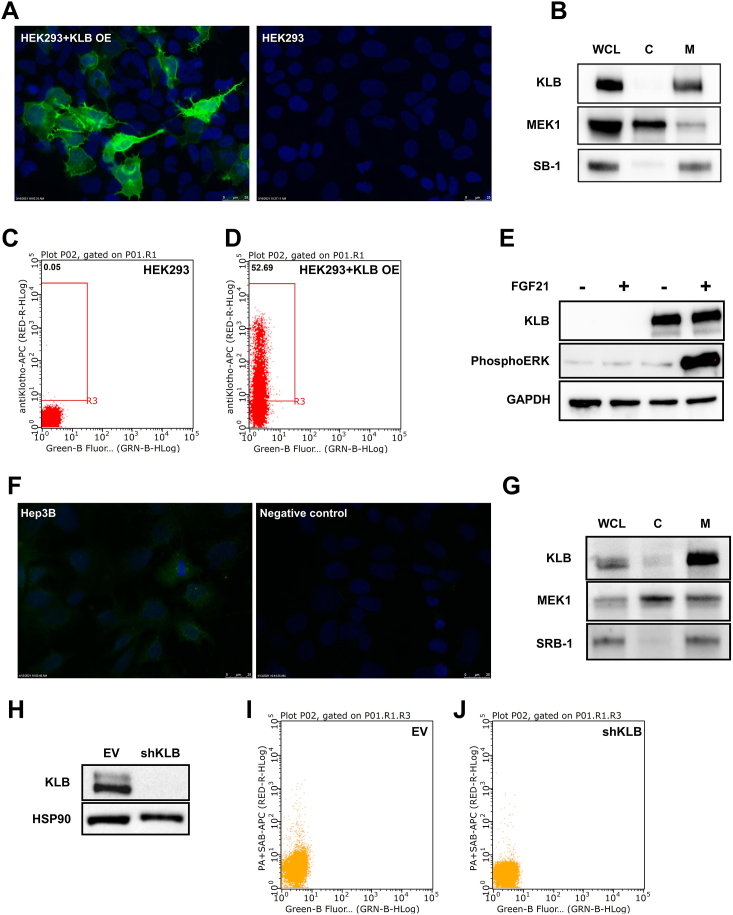
Subcellular localization of KLB in HEK293 cells with ectopic KLB expression and Hep3B cells with endogenous KLB expression. Localization of KLB expression in HEK293 cells using (A) IF, (B) immunoblot of KLB, MEK1/2, and SRB-1 after subcellular fractionation, (C and D) FACS analysis at 4 °C, and (E) immunoblot of KLB, GAPDH, and downstream phosphorylated ERK (phosphoERK) in response to FGF21. KLB localization in Hep3B cells using (F) IF, (G) immunoblot of KLB, MEK1/2, and SRB-1 after subcellular fractionation, (H) immunoblot of KLB and HSP90 to validate lentiviral knockdown of KLB in Hep3B cells, and (I and J) FACS analysis at 4 °C. All experiments were performed using one set of samples for each condition but reproducible in more biological replicates. C, cytoplasm; M, membrane; OE, overexpression; shKLB, short hairpin KLB; WCL, whole-cell lysate.

While KLB is generally considered a plasma membrane coreceptor for FGF19 and FGF21, direct localization studies are limited. Additionally, a previous study by Triantis et al. (2010) reported KLB to be primarily localized in the endoplasmic reticulum rather than the plasma membrane.^41^ To further elucidate the subcellular distribution of KLB, we transfected HEK293 cells with a plasmid encoding human KLB and performed IF microscopy using AB4. In KLB-transfected cells, we observed strong cytoplasmic and membranous staining, which was absent in empty vector (EV)–transfected cells (Figure 5A). We also observed staining of membrane protrusions within the plasma membrane, which is typically observed after staining plasma membrane proteins. To quantify the distribution of KLB in different cellular compartments, KLB-transfected HEK293 cells were subjected to centrifugation-based subcellular protein fractionation. Immunoblotting revealed significant enrichment of KLB in the membrane fraction, with minimal presence in the cytoplasmic fraction (Figure 5B). To specifically assess plasma membrane localization, we examined KLB-transfected cells using FACS analysis at 4 °C to prevent receptor internalization. KLB-transfected cells showed a marked increase in surface staining compared to EV-transfected cells (Figure 5C–D). Functional evidence for membrane-localized KLB was obtained by stimulating EV- and KLB-transfected cells with FGF21. KLB-transfected cells exhibited robust phosphorylation of downstream extracellular signal-regulated kinase 1/2 if treated with FGF21 (Figure 5E), indicating the presence of functional KLB at the cell surface.

To address potential mislocalization due to overexpression, we also investigated the subcellular localization of endogenous KLB in Hep3B cells. IF microscopy revealed weak cytoplasmic and membranous staining, which was absent in Hep3B cells stained with only a secondary antibody (Figure 5F). Subcellular fractionation and subsequent immunoblotting of Hep3B cells showed enrichment of KLB in the membrane protein fraction (Figure 5G). To confirm plasma membrane localization, we generated Hep3B cells with a stable KLB knockdown using shRNA (Figure 5H). FACS analysis at 4 °C demonstrated a reduced presence of KLB on the cell surface in cells with a KLB knockdown compared to control cells (Figure 5I–J), confirming the presence of endogenous KLB expression at the plasma membrane.

### KLB Protein Levels in Liver Biopsies from Patients with MASLD/MASH

**Figure 6:**
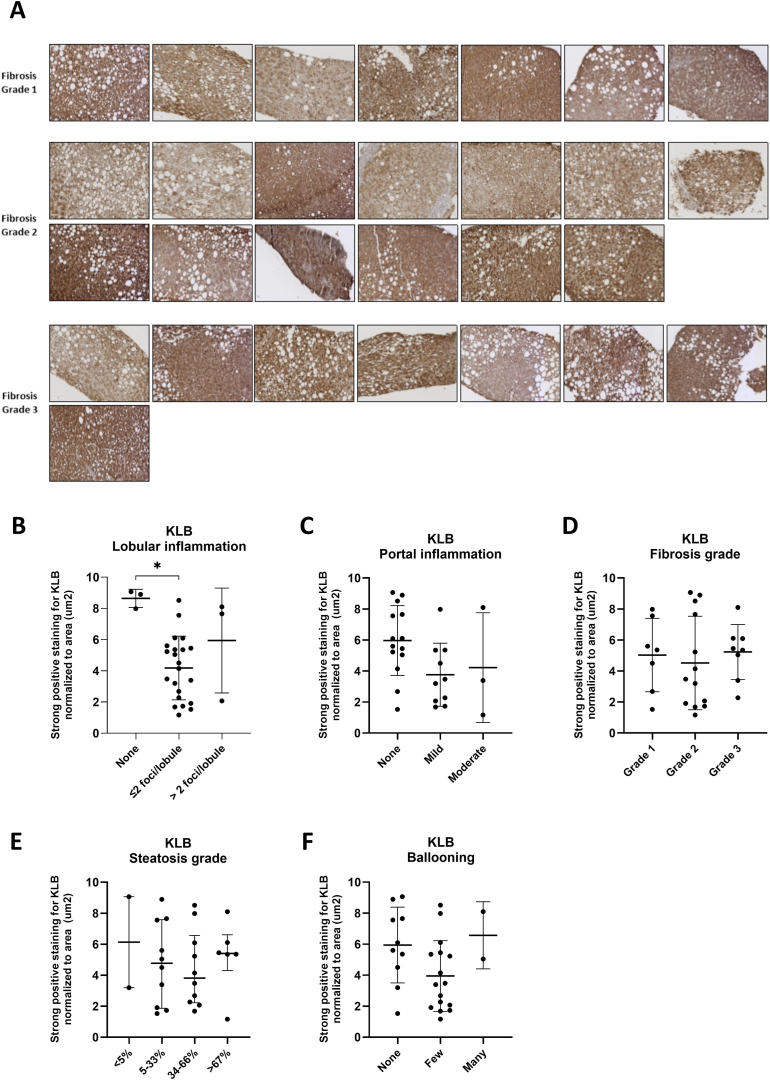
Quantified KLB protein levels using AB4 in liver biopsies of patients with varying stages of MASLD (n = 28). (A) Overview of patient liver biopsies with MASLD stained with AB4, separated into different fibrosis grades. (B and C) Association of KLB protein expression levels with typical MASLD characteristics, specifically (B) lobular inflammation (Kruskal–Wallis, *P* = .0168), (C) portal inflammation (Kruskal–Wallis, *P* = .0809), (D) fibrosis grade (Kruskal–Wallis, *P* = .6602), (E) steatosis grade (Kruskal–Wallis, *P* = .7834), and (F) ballooning (Kruskal–Wallis, *P* = .1097).

Previous research has indicated that pathological conditions can reduce KLB mRNA and protein levels, potentially limiting FGF activity.^42^ To assess whether hepatic KLB expression is reduced by MASLD disease activity, we performed IHC staining and quantified KLB protein levels in liver biopsies from patients with varying stages of MASLD/MASH. KLB protein expression was detected in all liver biopsies of patients with MASLD with considerable inter-individual variability (Table, Figure 6A). Overall, hepatic KLB expression was observed in liver biopsies from patients at both early and advanced MASLD/MASH disease stages. Hepatic KLB protein levels were inversely associated with lobular inflammation (*P* = .0168), showing significantly lower hepatic KLB at higher levels of lobular inflammation (Figure 6B). Hepatic KLB was not significantly associated with portal inflammation (*P* = .0809) but showed a trend with lower hepatic KLB protein at higher levels of portal inflammation (Figure 6C). No significant associations were observed between KLB protein levels and histology-derived fibrosis (*P* = .6602), steatosis (*P* = .7834), and ballooning scores (*P* = .1097), nor with MRI-derived proton density fat fraction (R = −0.1884, *P* = .3567), intravoxel incoherent motion diffusion (R = −0.1209, *P* = .5737), intravoxel incoherent motion fibrosis (R = 0.2009, *P* = .3466), and cT1 scores (R = −0.2185, *P* = .3166)(Figure 6D–F, Figure A10C–F). Further correlation analyses of hepatic KLB protein levels with other MASLD-related parameters revealed no significant correlations after correction for multiple testing, while aspartate aminotransferase (ASAT) levels did show a trend with lower hepatic KLB protein (R = −0.5881, *P* = .0526) (Figure A10A–B, Table A5).

**Table:** Clinical Characteristics of Patient Biopsies From the ANCHOR Study (n = 28)

Patient characteristics	N (%) or median (interquartile range)
Sex (male)	14 (50%)
Age (y)	47 (37; 53)
Ethnicity	
Western	20 (71%)
African	3 (11%)
Asian	3 (11%)
Other	2 (7%)
Height (cm)	174.5 (168; 183)
Weight (kg)	99.1 (90.5; 110.4)
Body Mass Index	33.0 (31.0; 35.2)
Waist circumference (cm)	114 (102; 119.3)
Hip circumference (cm)	112 (109; 118.5)
Diabetes mellitus type 2	9 (32%)
Hypertension (self-reported)	9 (32%)
Systolic blood pressure (mm/Hg)	133.5 (126; 142.3)
Diastolic blood pressure (mm/Hg)	85 (81; 90.5)
Liver parameters	
Fibrosis grade according to Brunt	
Grade 1 (perisinusoidal/pericellular fibrosis)	7 (25%)
Grade 2 (periportal fibrosis)	13 (46%)
Grade 3 (bridging fibrosis)	8 (29%)
Steatosis grade	
Grade 0 (<5%)	2 (7%)
Grade 1 (5%–33%)	10 (36%)
Grade 2 (34%–66%)	10 (36%)
Grade 3 (>67%)	6 (21%)
Lobular inflammation	
Grade 0 (no inflammation)	3 (11%)
Grade 1 (≤2 foci per lobule)	22 (78%)
Grade 2 (>2 foci per lobule)	3 (11%)
Portal inflammation	
None	11 (39%)
Mild	13 (46%)
Moderate	3 (11%)
Ballooning	
Grade 0 (normal hepatocytes)	10 (36%)
Grade 1 (clusters of hepatocytes with a rounded shape and pale cytoplasm, but of normal size)	16 (57%)
Grade 2 (presence of clusters of hepatocytes with at least one enlarged/ballooned hepatocyte)	2 (7%)
Medicine use	
Use of statins	7 (25%)
Use of PCSK9	1 (4%)
Use of metformin	8 (29%)
Use of gliclazide	5 (18%)
Use of betablockers	3 (11%)
Use of ACE inhibitors	4 (14%)
Use of ARB	4 (14%)
Use of calciumchannelblockers	2 (7%)
Use of proton pump inhibitors	7 (25%)
Plasma parameters	
Hemoglobulin (g/dL)	9.2 (8.4; 9.6)
Thrombosis	239 (221; 308)
Total cholesterol (mmol/L)	5.1 (4.5; 5.6)
LDLc (mmol/L)	2.9 (2.4; 3.8)
Triglycerides (mmol/L)	1.8 (1.2; 2.3)
Fasted glucose (mg/dL)	6 (5.6; 8.6)
Hba1c at baseline (mmol/mol)	40 (36; 51.5)
Fasted insulin (mIU/mL)	111 (64; 128)
Aspartate aminotransferase (U/L)	39 (33.5; 48.5)
Alanine aminotransferase (U/L)	57 (42; 69.8)
Atrial fibrillation	87.5 (67.8; 104.8)
Gamma-glutamyl transferase (U/L)	56 (29.5; 93.3)
Total bilirubin (mg/dL)	8 (7; 13.3)
Albumin (g/L)	47 (43.3; 48)
C-reactive protein (mg/L)	3.1 (2; 5.7)
Urine parameters	
Urine creatinine (mmol/L)	9.3 (5.9; 11)
Urine UMKR (mg/L)	1.4 (0.6; 2.7)

Data are given as absolute values (percentage of total) or as median (interquartile range) based on descriptive statistics. ACE, angiotensin-converting enzyme; ARB, angiotensin II receptor blocker; Hba1c, hemoglobin A1c; LDLc, low-density lipoprotein cholesterol; PCSK9, proprotein convertase subtilisin/kexin type 9; UMKR, urinary microalbumin/creatinine ratio.

## Discussion

In this study, we mapped KLB protein expression across various healthy human tissues and in liver biopsies from patients with varying stages of MASLD. We identified strong KLB expression in human liver, gallbladder, adipose tissue, pancreas, colon, and stomach. Additionally, we describe findings pointing towards KLB localization in the plasma membrane. Furthermore, while we established an association between KLB protein levels and hepatic lobular inflammation, overall hepatic KLB expression was not greatly affected by MASLD disease activity.

### KLB Antibody Validation

Although KLB protein expression data are available in the Human Protein Atlas database ^[dataset]^^38^, the employed antibody has been scored as uncertain for immunoblotting and IHC. When validated for immunoblotting, this antibody detected a strong 56–72 kDa band in human plasma, while no band was observed in human liver, where KLB is predominantly expressed. When validated for IHC, there was low consistency between antibody staining and mRNA expression data. To address this issue, we systematically validated the performance of four commercially available antibodies (AB1-4). While all ABs detected recombinant KLB, only AB4 correctly detected both recombinant and endogenous KLB in various antibody-based methods, such as immunoblotting, IHC, and IF. Hence, these observations emphasize that a thorough multistep validation process is crucial to determine antibody performance.

### KLB Protein Levels in Human Tissues

Our findings provide valuable information about the distribution of KLB in different tissues, which is crucial for understanding its physiological role and identifying potential target organs of FGF-based drugs. The strong expression of KLB in hepatocytes is in line with previous (pre)clinical studies implicating the involvement of KLB in liver metabolism and bile acid homeostasis. Multiple lines of evidence support a role for hepatic KLB in regulating FGF19-driven inhibition of bile acid synthesis through transcriptional repression of cholesterol 7-alpha hydroxylase, the rate-limiting enzyme of bile acid synthesis.^24^^,^^25^ Additionally, administration of FGF19 to mice and human liver organoids decreased de novo lipogenesis.^11^^,^^43^ Although KLB expression in the gallbladder has previously been documented,^44^ its specific function in the biliary tract remains unclear. While FGF19 is traditionally regarded as an ileal-derived plasma hormone, transcriptome data indicate a 20-fold higher FGF19 mRNA expression in the gallbladder compared to the small intestine ^[dataset]^.^38^^,^^44^^,^^45^ Similarly, FGF19 concentrations are 10–20 times higher in human bile than systemic circulation.^44^^,^^45^ Hence, the coexistence of KLB and FGF19 in the biliary tract suggests undiscovered functions of these proteins in regulating cholangiocyte activity. In support of this theory, *Klb*-deficient mice presented with reduced gallbladder size and resistance against gallstone formation.^24^

While our findings replicated previous findings of KLB expression in the liver and biliary tract, we also identified new expression sites. The expression of KLB in gastric glands and colonic intestinal glands, albeit weak, suggests a potential role in gastrointestinal function. This is particularly intriguing considering the dose-dependent gastrointestinal side-effects reported in clinical trials with FGF-based drugs, like abdominal pain and cramping, diarrhea, and nausea, which can impact patient compliance and treatment efficacy.^46^ While the exact underlying mechanisms of these side effects are not fully understood, off-target activation of KLB in mucus-producing intestinal cells may partially explain the reported discomfort. A better understanding of how KLB regulates the function of gastrointestinal glands could help mitigate these treatment-related gastrointestinal side effects in the future.

Detection of KLB in the adipose tissue is consistent with previously reported high mRNA expression levels. Especially during adipocyte differentiation, KLB expression was strongly increased. Moreover, treatment with FGF21 increased the ability of adipocytes to take up glucose and increased plasma levels of the adipose-derived hormone adiponectin in mice, primates, and humans.^47^^,^^48^ Additionally, preclinical research has demonstrated that the acute insulin-sensitizing effects of FGF21 are partially driven by KLB in adipose tissue.^20^^,^^21^ Collectively, these findings suggest direct targeting of KLB in adipose tissue.

While KLB is reportedly involved in both the exocrine and endocrine functions of the pancreas, we only detected KLB expression in pancreatic islets. Functions attributed to KLB in the endocrine pancreas are the control of insulin and glucagon secretion, and the protection of beta cells against glucolipotoxicity and apoptosis.^49^ Previously, beta cell-specific deletion of KLB was reported to modulate glucose-stimulated insulin secretion independent of FGF21.^50^ However, studies exploring the role of KLB in human beta cell models remain scarce.

Despite evidence of mRNA expression, the lack of KLB protein expression in mammary tissue, lung, and testis tissues in our study underscores the importance of protein-level validation. It is important to note that all tissue samples were stained using a single staining method. Hence, detecting KLB in tissues with low expression may require further optimization. Finally, our tissue profiling study also shows that KLB expression is strongly conserved between mice and humans, although evidence for central KLB expression in humans is still lacking.^22^^,^^51^^,^^52^

### Subcellular Localization of Ectopically and Endogenously Expressed KLB

While it is widely assumed that KLB is localized in the plasma membrane and functions as a coreceptor for FGF19 and FGF21, there is limited direct evidence supporting this hypothesis. Triantis et al. (2010) reported that KLB is predominantly localized in the ER rather than the plasma membrane.^41^ Therefore, we determined the subcellular distribution of both ectopically and endogenously expressed KLB using various experimental approaches. Our findings confirm that KLB is localized to the plasma membrane in both ectopic and endogenous contexts and support its proposed role as a coreceptor for FGF19 and FGF21. Therefore, future studies should investigate plasma membrane trafficking of KLB and its regulation under different physiological and pathological conditions.

### KLB Protein Levels in Liver Biopsies from Patients with MASLD/MASH

Previous studies reported reduced expression of KLB mRNA and protein levels in various pathological conditions, potentially limiting FGF activity.^42^ Most of these studies focused on the regulation of KLB in adipose tissue in the context of obesity; however, limited information is available on the potential modulation of hepatic KLB expression in the context of MASLD. Hence, we quantified KLB protein expression in liver biopsies from patients with varying stages of MASLD. We detected KLB in all liver biopsies, confirming the liver as a primary site of KLB expression. Notably, we observed that lower hepatic KLB protein levels were associated with higher levels of lobular inflammation. However, no significant association was observed between KLB protein levels and other key histological or MRI-derived features of MASLD, including steatosis, hepatocyte ballooning, or liver fibrosis. This suggests that KLB expression may be more sensitive to inflammation than other aspects of MASLD pathology. These observations align with previous findings that inflammatory cytokines, such as TNF-α and IL-1β, can reduce KLB expression in adipocytes and hepatocytes by activating JNK and NF-κB pathways.^53^, ^54^, ^55^ Conversely, *Klb* deficiency in mice was also associated with hepatic inflammation.^16^ Hence, additional studies need to determine whether changes in KLB expression precede or follow the progression of inflammation in MASLD. Our data suggest that KLB could also play a role in other liver diseases where inflammation plays a role, such as viral hepatitis. We did not find an association between KLB protein levels and fibrosis or steatosis grades. This could possibly be explained by the fact that we did not include severe cases of fibrosis and cirrhosis in this study. Moreover, we observed considerable inter-individual variability in hepatic KLB protein levels, suggesting that other factors may also influence KLB expression. This variability could affect patient responses to FGF-based therapies and warrants further investigations into other determinants of hepatic KLB expression. Similarly, KLB expression might also influence the treatment efficacy of other drugs. SGLT2 inhibitors, known for improving hepatic steatosis and fibrosis, have been reported to induce these beneficial effects through the enhancement of FGF-21 activity.^56^^,^^57^ Consequently, the efficacy of SGLT2 inhibitors might be affected by KLB expression levels.

While we performed correlation analysis on hepatic KLB protein levels and other clinical patient parameters related to MASLD, we found no significant associations with liver damage markers, plasma triglycerides, or plasma inflammatory markers after multiple testing correction. Nonetheless, we did observe a trend for plasma ASAT levels. A possible association with ASAT, a marker of liver injury, is supported by the findings that FGF-based therapeutics consistently cause potent reductions in the plasma levels of liver damage enzymes.^9^ In this study, we did not see an association of KLB protein levels with alanine transaminase levels. The lack of this association may suggest that KLB is not directly involved in hepatocellular injury, but rather reflects other aspects of liver pathophysiology, such as fibrosis progression or metabolic dysregulation.

## Conclusion

Our study provides new insights into the potential target organs of FGF-based therapeutics. The expression of KLB in the stomach and colon tissue could possibly underline the gastrointestinal side effects that are frequently reported with the use of FGF19/21 analogs. Although hepatic KLB expression correlated with inflammation, hepatic KLB expression remained relatively stable during MASLD disease progression. Overall, our findings support the use of FGF-based drugs in patients with early and advanced MASLD.
