# Supplementary material for: Beta-Klotho Protein Expression in Healthy Human Tissues and Liver Biopsies From Patients With MASLD or MASH
Source: Gastro Hep Adv. 2025 Jul 11;4(10):100745. doi: 10.1016/j.gastha.2025.100745 (PMC12421644; doi:10.1016/j.gastha.2025.100745)
Supplement: Supplementary Materials [file mmc1.docx]

**SUPPLEMENTARY MATERIAL**

**Table A1. Donor information of human paraffin tissue sections used for antibody validation.** All tissues were derived from the Biochain Institute.

| **Tissue name** | **Sex** | **Age** | **Clinical diagnosis** | **Catalog no.** | **Lot no.** |
| --- | --- | --- | --- | --- | --- |
| Liver | Male | 26 | Normal | T2234149 | C810124 |
| Spleen | Unknown | Unknown | Normal | GTX24372 | Absent |

**Table A2. Donor information of human total protein lysates used for antibody validation.**

| **Tissue name** | **Sex** | **Age** | **Clinical diagnosis** | **Catalog no.** | **Lot no.** |
| --- | --- | --- | --- | --- | --- |
| Liver | Male | 64 | Normal | P1234149 | C502292 |
| Spleen | Male | 78 | Normal | P1234246 | C104048 |

**Table A3. Donor information of human and monkey paraffin tissue sections used for IHC.** All tissues were derived from the Biochain Institute.

| **Tissue name** | **Organism** | **Sex** | **Age** | **Clinical diagnosis** | **Catalog no.** | **Lot no.** |
| --- | --- | --- | --- | --- | --- | --- |
| Testis | Human | Male | 60 | Normal | T2234260 | C404065 |
| Ileum | Human | Female | 79 | Diabetes | T2236227DIA | A911194 |
| Stomach | Human | Male | 36 | Normal | T2234248 | B903057 |
| Colon | Human | Male | 30 | Normal | T2234090 | C501483 |
| Lung | Human | Male | 24 | Normal | T2234152 | B911154 |
| Breast | Human | Female | 27 | Normal | T2234086 | B608235 |
| Pancreas | Human | Female | 66 | Normal | T2234188 | B908029 |
| Liver | Human | Female | 61 | Normal | T2234149 | C507040 |
| Duodenum | Human | Female | 64 | Diabetes | T2236101DIA | A711068 |
| Adipose | Human | Male | 26 | Normal | T2234003 | A808183 |
| Gallbladder | Human | Male | 25 | Normal | T2234118 | A603631 |
| Liver | Monkey | Female | 3 | Normal | T8534448CY | B903218 |

**Table A4. Primers used for qPCR analysis.**

| **Gene** | **Forward sequence (5’-3’)** | **Reverse sequence (5’-3’)** | **Company** |
| --- | --- | --- | --- |
| hKLB | TGTTCTTAAGCCCGAGTCTGT | AATCTGTGCATTGAGCGGGT | ThermoFisher Scientific |
| hU36B4 | TAGTTGGACTTCCAGGTCGC | CGTCCTCGTGGAAGTGACAT | ThermoFisher Scientific |

**Table A5. Spearman correlation analysis of hepatic KLB levels from IHC analysis of liver biopsies from patients with MASLD with all relevant histological and clinical parameters within the ANCHOR dataset (n=28).** Adjusted p-values are given after Benjamini-Hochberg was performed to correct for multiple testing. All correlations are given as r [95% CI] and marked with an asterisk (*) if significant (*P* ≤ .05) prior to multiple testing correction.

|  |  |  |
| --- | --- | --- |
| Basal patient characteristics | **Correlation as R [ 95% confidence interval ]** | **Adjusted *P* value after**  **Benjamini-Hochberg correction** |
| Sex ( male ) | 0.0742 [ -0.3108 ; 0.4383 ] | 1.0919 |
| Age ( years ) | 0.1484 [ -0.2414 ; 0.4970 ] | 1.2383 |
| Ethnicity | -0.2949 [ -0.6041 ; 0.0916 ] | .7229 |
| Height ( cm ) | -0.0190 [ -0.3925 ; 0.3599 ] | .9931 |
| Weight ( kg ) | 0.0678 [ -0.3166 ; 0.4330 ] | 1.0528 |
| BMI | 0.0961 [ -0.2908 ; 0.4559 ] | 1.3022 |
| Waist circumference ( cm ) | -0.2094 [ -0.5484 ; 0.1888 ] | .9973 |
| Hip circumference ( cm ) | -0.0283 [ -0.4069 ; 0.3586 ] | 1.0062 |
| Diabetes Mellitus Type 2 | 0.04336 [ -0.3385 ; 0.4129 ] | 1.0170 |
| Hypertension ( self-reported ) | -0.1214 [ -0.4760 ; 0.2671 ] | 1.3105 |
| Systolic blood pressure ( mm/Hg ) | 0.0735 [ -0.3114 ; 0.4377 ] | 1.0572 |
| Diastolic blood pressure ( mm/Hg ) | 0.3449 [ -0.3613 ; 0.6383 ] | .7028 |
| Liver parameters |  |  |
| Fibrosis grade | -0.1105 [ -0.4674 ; 0.2773 ] | 1.2562 |
| Steatosis grade | -0.0188 [ -0.3924 ; 0.3600 ] | .9689 |
| Lobular inflammation | 0.1270 [ -0.2618 ; 0.4804 ] | 1.3426 |
| Portal inflammation | -0.2903 [ -0.6059 ; 0.1044 ] | .7038 |
| Ballooning | -0.2086 [ -0.5423 ; 0.1820 ] | 1.0597 |
| Medicine use |  |  |
| Use of statins | 0,3412 [ -0.0403 ; 0.6359 ] | .5887 |
| Use of PCSK9 | -0.0119 [ -0.3931 ; 0.3728 ] | .9520 |
| Use of metformin | -0.1155 [ -0.5515 ; 0.3701 ] | 1.2177 |
| Use of gliclazide | 0.0227 [ -0.4608 ; 0.4958 ] | .9514 |
| Use of betablockers | -0.2572 [ -0.6001 ; 0.1656 ] | .9009 |
| Use of ACE inhibitors | -0.0908 [ -0.4787 ; 0.3267 ] | 1.1656 |
| Use of ARB | 0.0277 [ -0.3821 ; 0.4284 ] | .9895 |
| Use of calciumchannelblockers | -0.0818 [ -0.4716 ; 0.3348 ] | 1.1268 |
| Use of proton pump inhibitors | -0.0385 [ -0.4089 ; 0.3427 ] | 1.0113 |
| Plasma characteristics |  |  |
| Hemoglobulin ( g/dL ) | 0.0514 [ -0.3534 ; 0.4400 ] | 1.0642 |
| Thrombosis | 0.0838 [ -0.3246 ; 0.4658 ] | 1.1493 |
| Total cholesterol ( mmol/L ) | -0.1925 [ -0.5481 ; 0.2221 ] | 1.1184 |
| LDLc ( mmol/L ) | -0.3191 [ -0.6360 ; 0.0899 ] | .7847 |
| Triglycerides ( mmol/L ) | 0.0380 [ -0.3651 ; 0.4291 ] | .9963 |
| Fasted glucose ( mg/dL ) | -0.0467 [ -0.4362 ; 0.3575 ] | 1.0448 |
| Hba1c at baseline ( mmol/mol ) | -0.0124 [ 0.4158 ; 0.3951 ] | .9533 |
| Fasted insulin ( mIU/mL ) | -0.4685 [ -0.7301 ; -0.0872 ] | .3312 |
| Aspartate aminotransferase (ASAT)( U/L ) | -0.5881 [ -0.7957 ; -0.2570 ] | .0526 |
| Alanine transaminase (ALAT)( U/L ) | -0.3872 [ -0.6852 ; 0.0216 ] | .7809 |
| Atrial fibrillation (AF) | -0.0950 [ -0.4677 ; 0.3065 ] | 1.2752 |
| Gamma-glutamyl transferase (GGT)( U/L ) | -0.1866 [ -0.5376 ; 0.2195 ] | 1.0542 |
| Total bilirubin ( mg/dL ) | 0.1238 [ -0.2798 ; 0.4902 ] | 1.2566 |
| Albumin ( g/L ) | -0.0672 [ -0.4765 ; 0.3661 ] | 1.0650 |
| C-reactive protein (CRP)( mg/L ) | -0.0974 [ -0.4838 ; 0.3208 ] | 1.1750 |
| Urine parameters |  |  |
| Urine creatinine ( mmol/L ) | 0.0569 [ -0.3568 ; 0.4520 ] | 1.0661 |

**
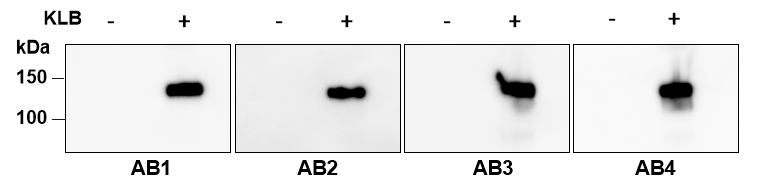
**

**Fig. A1. Detection of denatured recombinant human KLB.** Recombinant KLB expression was detected at an anticipated molecular weight of 100-150 kDa using AB1-4. The bars and numbers next to each panel indicate the position and size (kDa) of molecular markers, respectively.


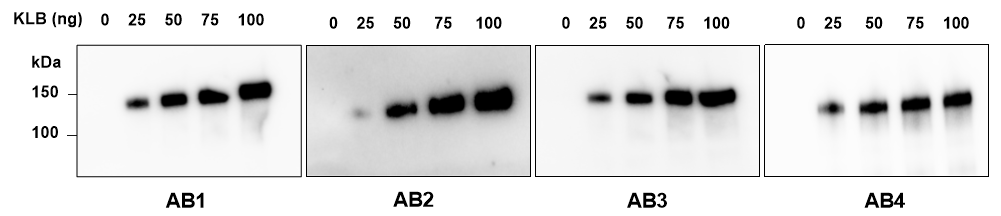


**Fig. A2. Dose-dependent detection of denatured recombinant human KLB.** Recombinant KLB was detected at an anticipated molecular weight of 100-150 kDa using AB1-4. The bars and numbers next to each panel indicate the position and size (kDa) of molecular markers, respectively.

**
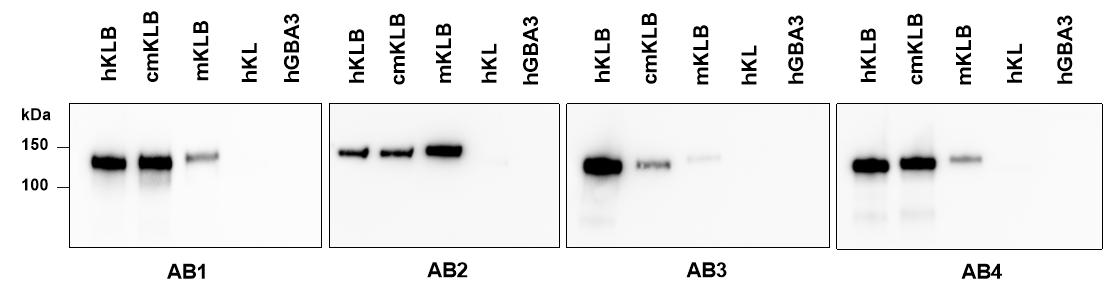
**

**Fig. A3. Determination of antibody cross-reactivity using recombinant human KLB, recombinant cynomolgus monkey KLB, recombinant mouse KLB, recombinant human Klotho (Kl), and recombinant human glucosylceramidase beta 3 (GBA3).** Cross-reactivity was determined by immunoblotting using AB1-4. The bars and numbers next to each panel indicate the position and size (kDa) of molecular markers, respectively.

**
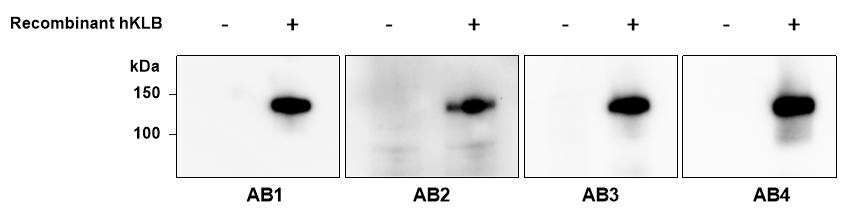
**

**Fig. A4. Assessment of antibody selectivity by spiking recombinant human KLB into a lysate of HEK293 cells**. KLB expression was detected at an anticipating molecular weight of 100-150 kDa by immunoblotting with AB1-4. The bars and numbers next to each panel indicate the position and size (kDa) of molecular markers, respectively.


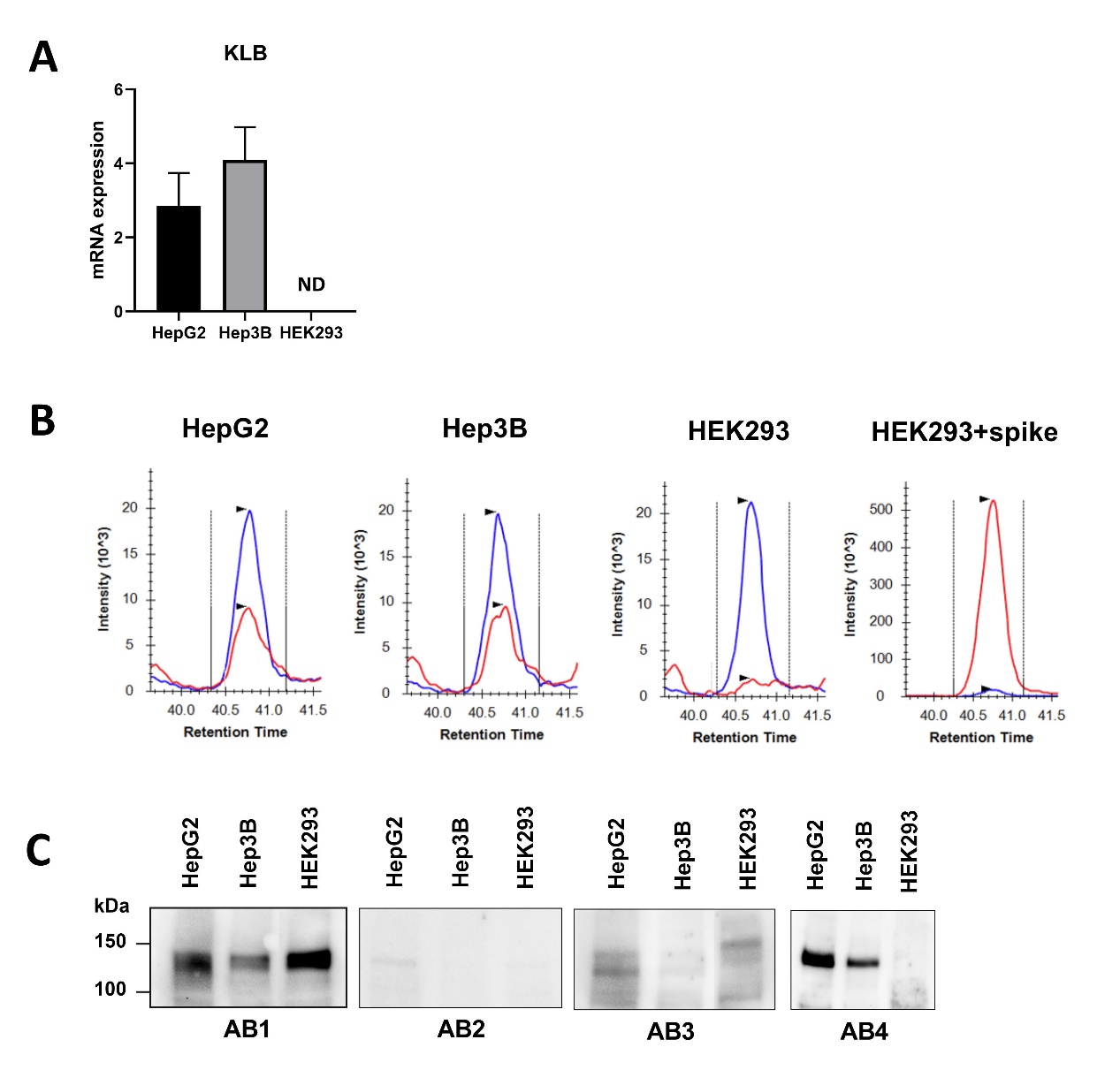


**Fig. A5. Detection of KLB in HepG2, Hep3B, and KLB-deficient HEK293 cell lines. (A)** mRNA gene expression of KLB, (**B**) targeted proteomics to detect KLB, and (**C**) KLB protein levels by immunoblotting with AB1-4, all performed in HepG2 and Hep3B cells with endogenous KLB expression, and HEK293 cells that lack endogenous KLB expression. The bars and numbers next to each panel indicate the position and size (kDa) of molecular markers, respectively.


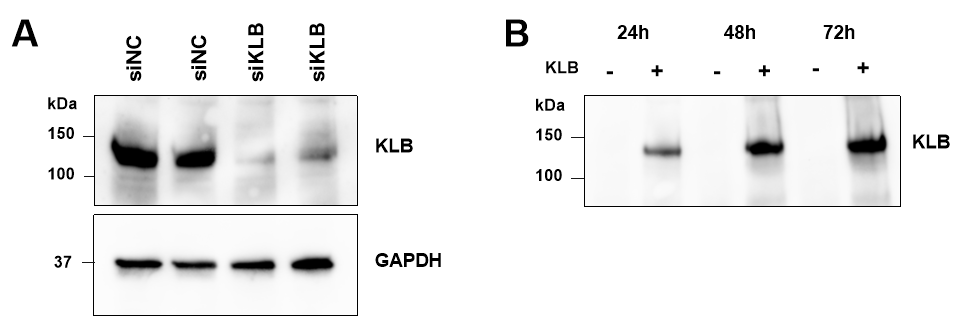


**Fig. A6. siRNA-mediated knockdown of KLB in Hep3B cells and KLB overexpression in HEK293 cells.** (**A**) KLB protein levels in Hep3B cells with endogenous KLB expression (siNC) and Hep3B cells with a siRNA-mediated knockdown of KLB (siKLB) using immunoblotting with AB4 and housekeeping protein GAPDH. (**B**) KLB protein levels in HEK293 cells were assessed by immunoblotting with AB4 following overexpression of human KLB at different time intervals. The bars and numbers next to each panel indicate the position and size (kDa) of molecular markers, respectively.


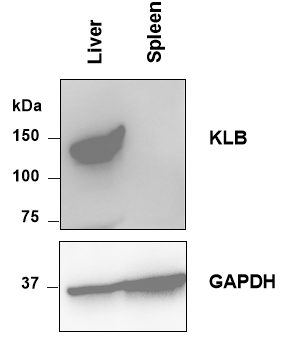


**Fig. A7. Detection of KLB in human liver and spleen protein lysates.** KLB was detected at an anticipated molecular weight of 100-150 kDa by immunoblotting with AB4. The bars and numbers next to each panel indicate the position and size (kDa) of molecular markers, respectively.


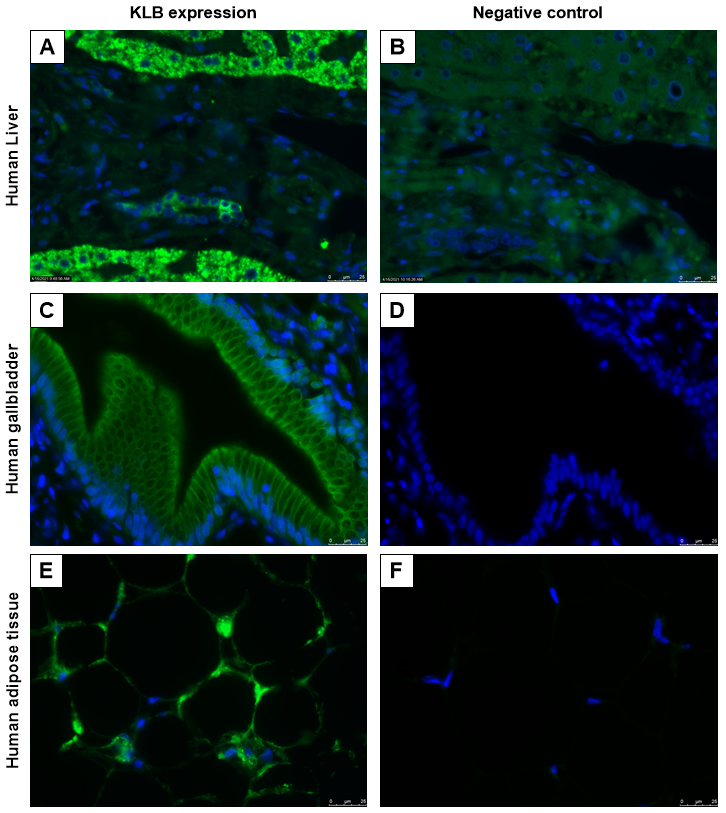


**Fig. A8. Detection of KLB expression in human tissues using immunofluorescence**. KLB expression and negative control by using only secondary antibody in human (**A-B**) liver, (**C-D**) gallbladder, and (**E-F**) adipose tissue. The scale bars of immunofluorescent images are 25 µm.

**
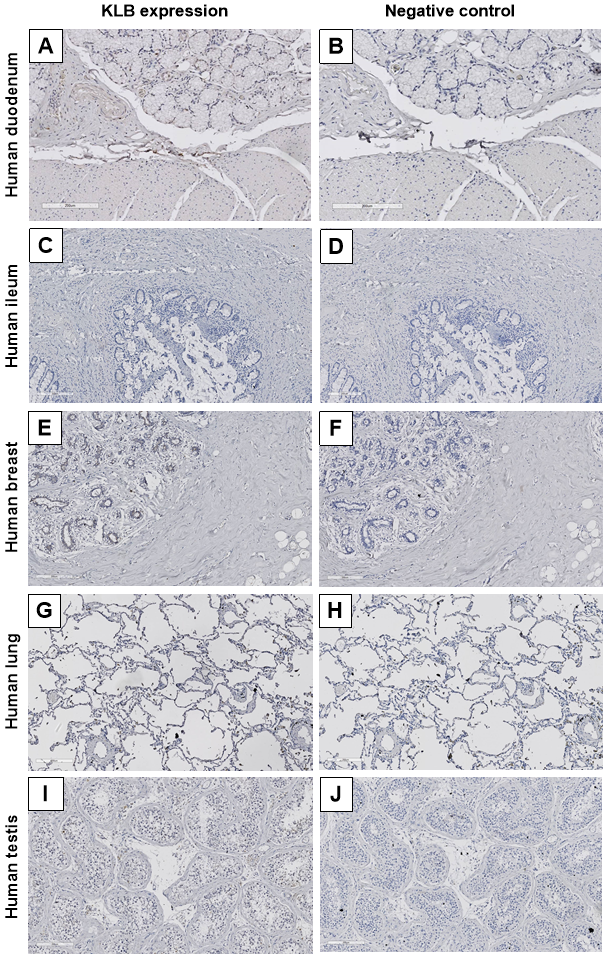
**

**Fig. A9. Detection of KLB expression in human small intestine, breast, lung, and testis.** KLB expression and negative control by only using secondary antibody in (**A-B**) human duodenum, (**C-D**) human ileum, (**E-F**) human breast tissue, (**G-H**) human lung tissue, (**I-J**) human testis tissue, through immunohistochemistry using AB4. The scale bars represent 200 µm.


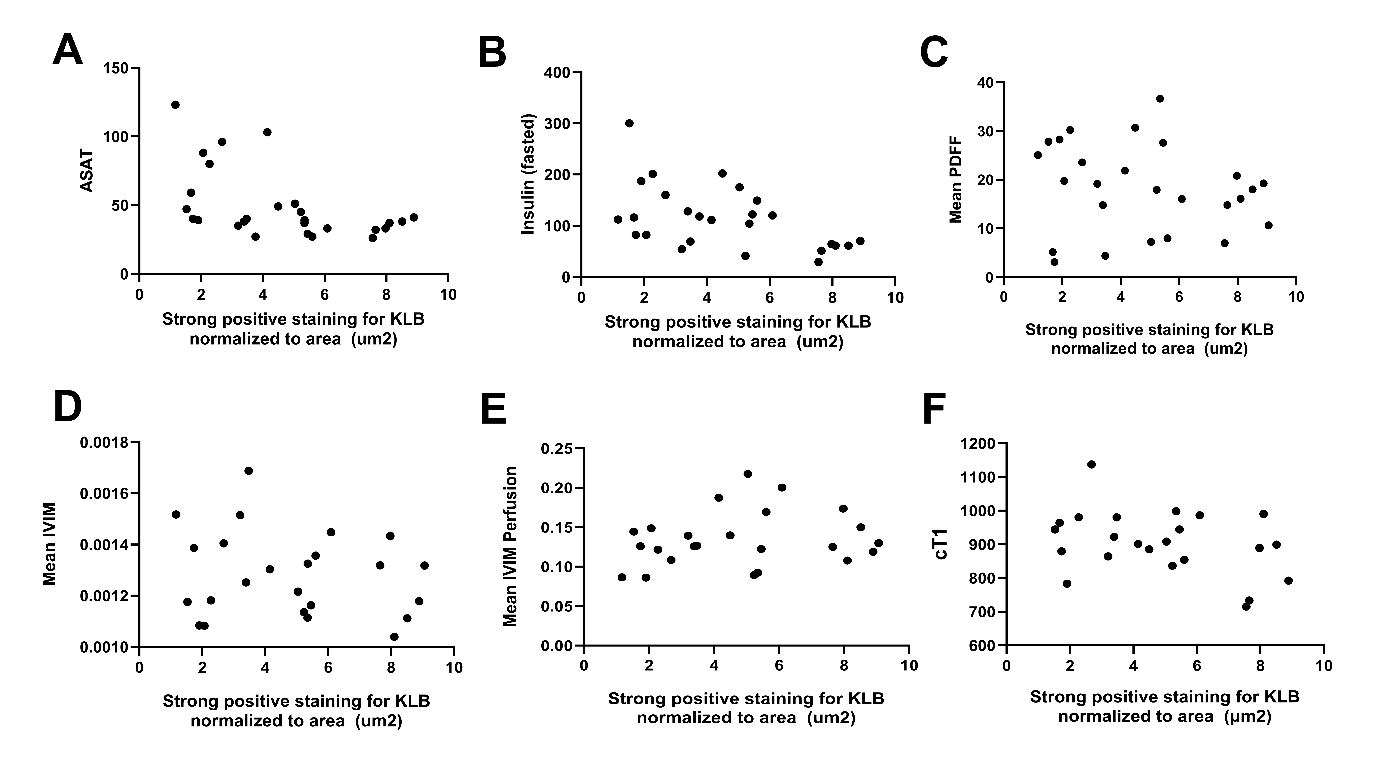


**Fig. A10. Spearman correlation analysis of KLB protein expression with clinical patient characteristics and MRI analyses of liver fat and fibro-inflammation in liver biopsies of patients with various stages of MASLD.** Correlation analysis of KLB protein expression with (**A**) ASAT levels (Spearman rho, R=-0.5881, *P*=.0526), (**B**) fasted insulin levels (Spearman’s rho, R=-0.4685, *P*=.3312), (**C**) mean PDFF (Spearman’s rho, R=-0.1884, *P*=.3567), (**D**) mean IVIM-D (Spearman’s rho, R=-0.1209, *P*=.5737), (**E**) mean IVIM perfusion score (Spearman’s rho, R=0.2009, *P*=.3466), and (**F**) corrected T1 levels (Spearman’s rho, R=-0.2185, *P*=.3166). PDFF, proton-density fraction. IVIM-D, intravoxel incoherent motion diffusion. IVIM-f, intravoxel incoherent motion fibrosis. cT1, corrected T1.

**SUPPLEMENTARY METHODS**

**MRI analyses of patients with MASLD**

Participants underwent a single-session liver MRI scan at a 3T MR scanner (Ingenia, Phillips). A magnitude-based PDFF scan was conducted with a multi-slice multi-echo gradient echo sequence. Prominent vessels, bile ducts, liver edges, and image artifacts were avoided in all post-processing steps. For PDFF, three circular regions of interest (ROIs) were placed in the liver on three different slices on the first echo time image. A multi-echo, multi-frequency water and fat signal model (including correction for T2* effects) was fitted (Matlab version 2021b; The Mathworks Inc.) to the mean signal intensity in the three ROIs per echo time to obtain liver fat fraction (PDFF) and T2* value, as described previously (Yokoo et al., 2011). and T2* value, as described previously. The LiverMultiScan® software was used to perform cT1 mapping of the liver. Disease activity was quantified using intravoxel incoherent motion (IVIM) imaging. The IVIM sequence consisted of a free-breathing multislice diffusion weighted single-shot echo-planar imaging sequence with 18 unique b-values.

**Cell lines**

HEK293 and HepG2 (CRL-1573 & HB-8065, ATCC) were cultured using Dulbecco’s Modified Eagle Medium (DMEM) GlutaMAX supplemented with 10% foetal bovine serum (FBS) and 1% penicillin/streptomycin (P/S). Hep3B cells (ATCC, HB-8064) were cultured using DMEM GlutaMAX supplemented with 10% FBS, 1% P/S, and 1.5 µg/mL Puromycin (ant-pr, Invivogen). HEK293 cells were transfected for 24 hours with 100 ng KLB vector (OHu24417, GenScript) and Fugene HD Transfection Reagent (E2311, Promega) in OptiMEM reduced medium (31985062, ThermoFisher Scientific).

**Lentiviral shRNA knockdown**

Stable short-hairpin RNA (shRNA)-mediated knockdown of human KLB (NM_175737.4) in Hep3B cells that endogenously express KLB was performed using 5’ GGATGAGAAAGAAGTTGTTCT 3’ as a targeting sequence in the shRNA template containing antisense and sense sequences, a hairpin loop sequence and restriction site overhangs. The designed shRNA was cloned into pLKA-TRC (10878, Addgene) and transfected into HEK293T (CRL-11268, ATCC) cells using polyethyleneimine together with packaging plasmid pMDLg/pRRe (12251, Addgene) and pRSV-Rev (12253, Addgene) and envelope plasmid pHCMV-G (75497, ATCC). Empty pLKO-TRC (10878, Addgene) was used as a control. Viral particles were harvested 48 hours after transfection. After addition of polybrene, the virus containing shRNA or empty virus was filtered and administered to wild-type Hep3B cells. Antibiotic selection started 48 hours after transduction by culturing cells in puromycin-containing culture medium (1.5 µg/mL puromycin).

**siRNA transfection**

siRNA-lipofectamine complexes by diluting 6 pmol siRNA for KLB (SASI_Hs01_00083505, Sigma Aldrich) and lipofectamine RNAiMAX (13778030, ThermoFisher Scientific) in Opti-MEM reduced serum medium. The siRNA-lipofectamine mixture was then added for 48 hours to Hep3B cells at a 50% confluency, giving a final concentration of 10 nM siRNA.

**Subcellular fractionation**

Subcellular protein fractions from HEK293 and Hep3B cells were obtained using a centrifugation-based subcellular fractionation kit (78840, Thermo Scientific). Cells were collected by centrifugation at 500xg for 5 minutes at 4°C, after which the cytoplasmic and membrane extract were collected according to the manufacturer’s protocol. All extraction buffers were supplemented with 1:100 Halt protease inhibitor cocktail (87785, Thermo Scientific).

**Immunofluorescence microscopy**

HEK293 cells were cultured in a monolayer on a Poly-L-Lysine 12 mm coverslip (354085, Corning) and fixated using 4% paraformaldehyde (PFA)(4078.9010, Klinipath). Cells were permeabilized with 0.1% Triton-X100 (T9284, Sigma Aldrich) in PBS for 30 minutes at 37°C, followed by blocking with 1% BSA for 30 min at RT. Each coverslip was incubated with 1:100 diluted goat anti-KLB antibody (AF5889, R&D Systems) in 1% BSA overnight at 4°C. Next, coverslips were washed and incubated with 1:400 diluted Alexa Fluor 488 donkey anti-goat IgG secondary antibody (A-11055, ThermoFisher Scientific) in 1% BSA and 1:500 diluted DAPI (60005620, Roche) in 1% BSA simultaneously for 1h at RT. Coverslips were mounted on a glass slide using mounting medium (S3023, Dako) and imaged on a Leica fluorescence microscope using a 40x oil objective. Images were analysed using Zenn 3.8 software.

**Fluorescence-Associated Cell Sorting (FACS)**

Hep3B and KLB-transfected HEK293 cells were suspended in cold DMEM culture medium with 10% FBS and 1% P/S, followed by incubation with a 1:100 dilution of KLB primary antibody (MAB5889, R&D Systems) for 40 minutes at 4°C. Cells were washed and then resuspended in cold DMEM culture medium with 10% FBS and 1% P/S. Next, cells were incubated with a 1:200 dilution of allophycocyanin-conjugated anti-rabbit IgG secondary antibody (F0111, R&D Systems) for 40 minutes at 4°C. Cells were washed again with cold PBS and then resuspended in cold PBS, followed by measurement at excitation wavelength 620-650 nm and emission wavelength 660-670 nm.

**Quantitative real-time PCR**

Total RNA was isolated from the cells using Tri reagent (T9424, Sigma Aldrich) following the manufacturer’s protocol. 1000 ng of isolated RNA was reverse transcribed into complementary DNA using 200 U/µL Moloney Murine Leukemia Virus reverse transcriptase (28025013, Invitrogen), 50 µM random nonamers (R7647, Sigma Aldrich), 10 mM deoxynucleotide triphosphates (11969064001, Roche), 40 U/µL RNAse Out (10777019, Invitrogen), and 0.1 M dithiothreitol (DTT)(28025013, Invitrogen) in buffer solution. cDNA was amplified using Hi-ROX SensiMix™ SYBR green (QT605-05, Bioline). Forward and reverse primers of KLB and housekeeping gene U36B4 were added in a final concentration of 300 nM (Table S4). qPCR analysis was performed on the Quantstudio5™ Real-Time PCR System (Applied Biosystems). Gene expression levels were quantified using the standard curve and normalized to the housekeeping gene.

**Immunoblotting**

Cell lysates were isolated using 0.1% NP40 buffer, composed of 0.4M NaCl, 1:1000 NP40, 10 mM Tris (pH=8.0), 1 mM EDTA (pH=8.0), 1x Complete protease inhibitor (11836145001, Roche), and 1:100 phosphatase inhibitor (78420, ThermoFisher Scientific). Protein concentrations were determined using the Pierce^TM^ BCA Assay kit (23225, Thermo Scientific). 30 µg protein was loaded per lane on a 4-15% precast polyacrylamide gel (4561084, Biorad) and gels were run at 130V. Samples were transferred to 0.2µm nitrocellulose membranes (1704159, Biorad) using the Trans-Blot Turbo transfer system (Biorad). Membranes were blocked in 5% BSA for 1h at RT and incubated overnight at 4°C with 1:1000 diluted KLB AB1 (106794, Abcam), AB2 (SAB2108630, Sigma Aldrich), AB3 (MAB5889, R&D systems), or AB4 (AF5889, R&D sytems), mouse anti-phospho-p44/42 MAPK (9106S, Cell Signaling), and mouse anti-GAPDH (CB1001, Calbiochem). Membranes were washed with tris-buffered saline supplemented with Tween-20 (TBS-T)(p2287, Sigma Aldrich), followed by 1h incubation with secondary antibodies rabbit anti-goat IgG-HRP (P0449, Dako), goat anti-rabbit IgG-HRP (P0448, Dako), and rabbit anti-mouse IgG-HRP (P0260, Dako). After washing with TBS-T, proteins were visualized using Supersignal West Pico Plus ECL mix (34578, Thermo Scientific) and analysed using Image Lab software version 6.0 (Bio-Rad).

**Targeted proteomics**

In-gel digestion was done on the excised gel bands between 100-200 kDa of the cell lysates (containing an equivalent of 600 000 cells) as described previously. Liquid chromatography (LC) was performed on a nano-ultra high-performance liquid chromatography (UHPLC) system (Ultimate UHPLC focused, Dionex, Thermo Fisher Scientific) to separate the peptides using a nanocolumn (Acclaim PepMap100 C18, 75 µm × 500 mm x 2 µm, 100 Å) with a linear gradient from 3% to 40% v/v acetonitrile containing 0.1% v/v formic acid over 90 minutes at a flow rate of 300 nL/min. The target peptides were analyzed using a triple quadrupole mass spectrometer (MS) equipped with a nano-electrospray ion source (TSQ Altis, Thermo Scientific). For the LC-MS measurements, digested peptides equivalent to 5% of the digested starting material were injected with 50 pg isotopically labeled ^13^C^15^N-lysine standard peptide (Pepotec Grade 2, Thermo Fisher Scientific). LFPDGIVTVANAK was used for the detection of KLB protein levels in cell lines. MS traces were manually curated using the Skyline software before integrating the peak areas and the examples of the detected peaks were visualized in the figure.
